# N terminal prohormone of brain natriuretic peptide is associated with improved heart rate recovery after treadmill exercise test

**DOI:** 10.1016/j.ijcrp.2023.200203

**Published:** 2023-08-17

**Authors:** Yi-Ting Lin, Lian-Yu Lin, Kai-Jen Chuang

**Affiliations:** a School of Medicine, College of Medicine, Taipei Medical University, Taipei, Taiwan; b Department of Internal Medicine, College of Medicine, National Taiwan University, Taipei, Taiwan; c Cardiovascular Center, National Taiwan University Hospital, Taiwan; d Department of Public Health, School of Medicine, College of Medicine, Taipei Medical University, Taipei, Taiwan; e School of Public Health, College of Public Health and Nutrition, Taipei Medical University, Taipei, Taiwan

**Keywords:** Brain natriuretic peptide, N terminal prohormone of brain natriuretic peptide, Heart rate recovery

## Abstract

**Background:**

Heart rate recovery (HRR) and N terminal-pro B type natriuretic peptide (NT-proBNP) are markers for survival and cardiac function; however, Little is known about their association.

**Method:**

We examined 2540 healthy subjects aged 12–49 years with data from National Health and Nutrition Examination Survey(NHANES) 1999–2002. HRR parameters 1–3 min after exercise were calculated from exercise test results. Baseline characteristics, anthropometric and NT-proBNP, and other risk covariates were obtained.

**Result:**

The results showed that NT-proBNP was positively correlated with HRR2(correlation coefficient (cc) = 0.042 [0.029–0.054], P < 0.001) and HRR3(cc = 0.046 [0.029–0.064], P = 0.001); with further adjustment, the associations remained significant between NT-proBNP and HRR2(cc = 0.030 [0.010–0.049], P = 0.004)/HRR3(cc = 0.029[0.004–0.054], P = 0.025). Our study also found significant correlations between NT-pro BNP and SBP(cc = −0.026 [-0.046∼-0.005], P = 0.017), DBP(cc = −0.037 [-0.062∼-0.012], P = 0.005), and total cholesterol(cc = −0.065 [-0.12∼-0.018], P = 0.009).

**Conclusions:**

Our results suggest that BNP might reduce heart rate after exercise by inhibiting the sympathetic nervous system (SNS), reducing HRR2 and HRR3, as these phases involve the reduction of heart rate through cardiac sympathetic withdrawal. Moreover, the interaction of BNP on the SNS might be related to the effect of BNP on cardiovascular risks.

## Highlights

• Little is known about the association between Heart rate recovery (HRR) and N terminal-pro B type natriuretic peptide (NT-proBNP).
• We examined 2540 healthy subjects with data from National Health and Nutrition Examination Survey(NHANES) 1999-2002.
• Positive correlation was found between NT-proBNP and heart rate recovery after exercise.
• We hypothesized NT-proBNP might reduce heart rate after exercise by inhibiting the sympathetic nervous system, thus affecting cardiovascular risks.

## 1 Introduction

Heart rate recovery (HRR) is the reduction in heart rate at peak exercise and the rate measured after a fixed-duration cool-down period [1].This method is commonly employed to evaluate cardiac autonomic function across different patient groups [[2], [3], [4], [5]]. Moreover, impaired HRR following exercise has been firmly established as a predictor for mortality, even in healthy individuals [1].

Brain natriuretic peptide(BNP) primarily originates from the heart and functions as a cardiac hormone [6]. It exerts systemic biological effects such as peripheral vasodilation, increased natriuresis, and diuresis, as well as inhibition of the sympathetic nervous system (SNS) and the renin-angiotensin-aldosterone system (RAAS) [[6], [7], [8]]. The N-terminal prohormone of brain natriuretic peptide (NT-proBNP) is a prohormone with a 76 amino acid N-terminal inactive protein cleaved from the molecule to release brain natriuretic peptide. The biological half-life of NT-proBNP is longer than that of BNP, making the peptide a better target than BNP for diagnostic blood testing. BNP and NT-proBNP are common and vital markers for diagnosing heart failure (HF) and cardiac dysfunction [[9], [10], [11]]. In recent times, numerous forensic investigations have shown that BNP and NT-proBNP could be used to reflect the cardiac function of patients before their death [12].

A high BNP in most circumstances is a sign of cardiac diseases; however, considering its biological effects, such as vasodilation and suppressing cardiac sympathetic nerve activity, we hypothesize that a higher BNP level might be associated with a better HRR value in relatively healthy subjects. We used data from National Health and Nutrition Examination Survey(NHANES)1999–2002 and investigated the association between HRR and NT-proBNP in participants with relatively healthy conditions.

## 2 Methods

### 2.1 Data source

NHANES is a survey that collects information about the health and nutrition of the civilian noninstitutionalized population in the United States. It used to be conducted periodically but became an annual survey in 1999. The survey data is released every two years, and online resources are available, such as survey operation manuals, consent documents, and brochures for NHANES 1999–2002 [13]. (https://www.cdc.gov/nchs/nhanes/about_nhanes.htm).

### 2.2 Study population

Between 1999 and 2002, a total of 21,004 participants were surveyed. The cardiovascular fitness test was applied to individuals aged between 12 and 49. Within this scope, 10,210 candidates were involved. However, out of these candidates, 7247 could not undergo the cardiovascular fitness test due to various medical reasons such as existing medical conditions, the influence of certain medications on heart rate, physical limitations, and restrictions related to heart rate, blood pressure, and irregular heart rhythms.

**Fig. 1:**
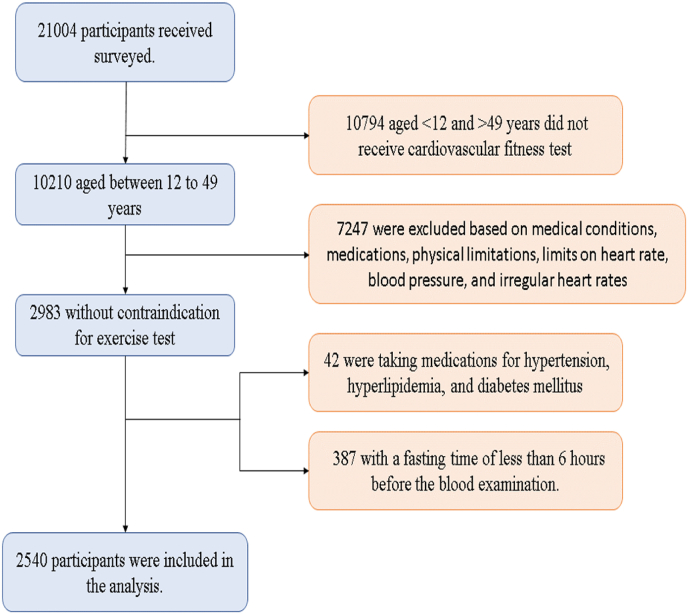
A flow diagram with inclusion and exclusion criteria to form the study population.

Among the initial group, 2963 participants aged 12 to 49 successfully underwent treadmill exercise tests, providing the necessary data for deriving HRR values. Of the 2963 participants, 42 subjects taking medications for hypertension, hyperlipidemia, and diabetes mellitus were excluded. Additionally, 387 subjects were further excluded from the analysis due to fasting times of less than 6 h before the blood examination. Consequently, the final sample for detailed analysis comprised 2540 subjects. The patient inclusion process is visually depicted in Fig. 1 through an illustrative patient inclusion flowchart.

### 2.3 HRR and treadmill test

The protocol for the treadmill test consisted of a 2-min warmup, two 3-min exercise stages, and a 2-min cool-down period [14]. The objective of this test was to induce a submaximal exercise effect, where the heart rate (HR) reached approximately 75% of the age-predicted maximum (220 minus age) by the conclusion of the test. HR was continuously monitored using an automatic device and recorded at the end of each stage. The 1-min HRR (HRR1) was calculated as the peak HR during the exercise test minus the HR at 1 min of recovery. Similarly, the 2-min HRR (HRR2) and 3-min HRR (HRR3) were calculated as the peak HR during the exercise test minus the HR at 2 and 3 min of recovery, respectively.

### 2.4 Measures of NT-proBNP

NT-proBNP (Roche Diagnostics) was measured in serum on the Roche Cobas e601 autoanalyzer. The lower and upper limits of detection are (5 pg/mL, 35000 pg/mL).CVs: 3.1% (low, 46 pg/mL) and 2.7% (high, 32805 pg/mL). The NT-proBNP result is expressed as pg/ml [15].

### 2.5 Anthropometric and biochemical data

Data were collected at all study sites by trained personnel. Sociodemographic information such as age, gender, and race was self-reported during the household interview. Laboratory measurements were performed in a mobile examination center. Weight and height were measured using standard methods and digitally recorded. Body mass index (BMI) was calculated as weight (Kg) divided by the square of height (m). Waist circumference was measured at the iliac crest to the nearest 0.1 cm. Three and sometimes four blood pressure were measured and averaged using a mercury sphygmomanometer.

Blood samples were processed and stored locally and were shipped to central laboratories for analysis. The smoking status was determined by measuring serum cotinine concentrations (ng/mL) and classified into four groups: non-smoker (<14 ng/mL), light smoker (14–99 ng/mL), moderate smoker (100–199 ng/mL), and heavy smoker (≥200 ng/mL) [16]. Renal function was represented by estimated glomerular filtration rate(eGFR), derived from the following formula: eGFR = 175 x (SCr)-1.154 x (age)-0.203 x 0.742 [if female] x 1.212 [if Black] [17,18].

The serum glucose level was measured by the hexokinase enzymatic reference method (COBRAS MIRA; Roche Diagnostics, Indianapolis, IN). Serum triglycerides, total cholesterol were measured enzymatically, and high-density lipoprotein (HDL) was measured using precipitation.

### 2.6 Data analysis

Data were expressed as mean ± S.E. Multiple linear regression models estimated the association magnitude between each HRR parameter and the serum NT-proBNP level. We used an extended model approach for linear regression models to adjust potential confounders. Model 1 was adjusted for age and gender. Model 2 was adjusted for model plus race, smoking status, BMI, waist, SBP, DBP, glucose, total cholesterol, HDL, triglyceride, eGFR and maximum heart rate increase during exercise. The correlation coefficients (B coefficients) and 95% confidence interval were reported. A p < 0.05 was considered statistically significant. Sampling weights for unequal selection probabilities, oversampling, and nonresponse were applied for all analyses using the Complex Sample Survey module of SPSS 20.0 for Windows XP (SPSS Inc. Chicago, Illinois, U.S.

## 3 Results

The basic characteristics of the subjects are demonstrated in Table 1. The mean age was 26.1 ± 0.3 years, and 68% were male gender. Most of the subjects (67.5%) were white people. The other characteristics, such as smoking status, measurements, and blood examinations, were also shown in Table 1.

**Table 1:** Characteristics of subjects for analysis.

Baseline characteristics	N = 2540
Age, years	26.1 ± 0.3
Male gender, %	68.0
Race, %	
Hispanic	17.3
Non-hispanic white	67.5
Non-hispanic black	9.9
Others	5.2
Smoking	
Non-smoker	75.3
Light smoker	7.0
Moderate smoker	8.7
Heavy smoker	9.0
**Measurement**	
BMI, kg/m^2^	25.18 ± 0.17
Waist, cm	86.63 ± 0.45
SBP, mmHg	112.95 ± 0.34
DBP, mmHg	69.01 ± 0.29
**Blood examination**	
Glucose, mg/dL	86.95 ± 0.45
Total cholesterol, mg/dL	181.38 ± 0.88
HDL, mg/dL	49.51 ± 0.52
Triglyercide, mg/dL	110.80 ± 2.64
eGFR, mL/min/1.73m^2^	122.70 ± 1.23
NT-proBNP, pg/mL	39.72 ± 1.26

Abbreviations. BMI, bod mass index; SBP, systolic blood pressure; DBP, diastolic blood pressure; HDL, high density lipoprotein; eGFR, estimated glomerulus filtration rate; NT-proBNP, N-terminal prohormone of brain natriuretic peptide.

The correlation between HRR and the blood NT-proBNP level is shown in Table 2. In model 1, adjusted for only age and gender, the blood NT-proBNP level was not significantly associated with the 1-min HRR (correlation coefficient (cc) = 0.009 [0.003–0.021], P = 0.149) but was significantly associated with the 2-min HRR (cc = 0.042 [0.029–0.054], P < 0.001) and the 3-min HRR (cc = 0.046 [0.029–0.064], P < 0.001). In the fully adjusted model, the blood NT-proBNP level was not significantly associated with the 1-min HRR (cc = 0.013 [−0.008∼0.034], P = 0.215) but was significantly associated with the 2-min HRR (cc = 0.030 [0.010–0.049], P = 0.004) and the 3-min HRR (cc = 0.029 [0.004–0.054], P = 0.025).

**Table 2:** Correlation coefficients of regression analysis between heart rate recovery and blood N-terminal prohormone of brain natriuretic peptide level.

	HRR1	P	HRR2	P	HRR3	P
Model1						
NT-proBNP	0.009 [-0.003-0.021]	0.149	0.042 [0.029–0.054]	<0.001	0.046 [0.029–0.064]	<0.001
Model2						
NT-proBNP	0.013 [-0.008∼0.034]	0.215	0.030 [0.010–0.049]	0.004	0.029 [0.004–0.054]	0.025

Model 1: adjusted for age and gender.

Model 2: adjusted for Model 1 plus race, smoking status, BMI, waist, SBP, DBP, glucose, total cholesterol, HDL, triglyceride, eGFR and maximum heart rate increase during exercise.

Abbreviations; HRR1, 1-min heart rate recovery; HRR2, 2-min heart rate recovery; HRR3, 3-min heart rate recovery; NT-proBNP, N-terminal prohormone of brain natriuretic peptide.

The associations between the blood NT-proBNP level and the risk factors are demonstrated in Table 3. As shown in Table 3, the blood NT-proBNP level had a significant inverse correlation with the SBP (cc = −0.026 [−0.046∼-0.005], P = 0.017), the DBP (cc = −0.037 [−0.062∼-0.012], P = 0.005) and the blood total cholesterol level (cc = −0.065 [−0.12∼-0.018], P = 0.009). The blood NT-proBNP level was not significantly associated with the blood glucose, HDL, and triglyceride level.

**Table 3:** Correlation coefficients of regression analysis between the blood N-terminal prohormone of brain natriuretic peptide level with the risk factors.

	NT-proBNP	P
SBP	−0.026 [-0.046∼-0.005]	0.017
DBP	−0.037 [-0.062∼-0.012]	0.005
Glucose	0.013 [-0.014∼0.039]	0.329
Total cholesterol	0.065 [-0.12∼-0.018]	0.009
HDL	−0.002 [-0.026∼0.023]	0.894
Triglyceride	−0.037 [0.195–0.121]	0.635

Model adjusted for age, gender, race, smoking status, BMI, waist and eGFR.

SBP, systolic blood pressure; DBP, diastolic blood pressure; HDL, high density lipoprotein.

**Fig. 2:**
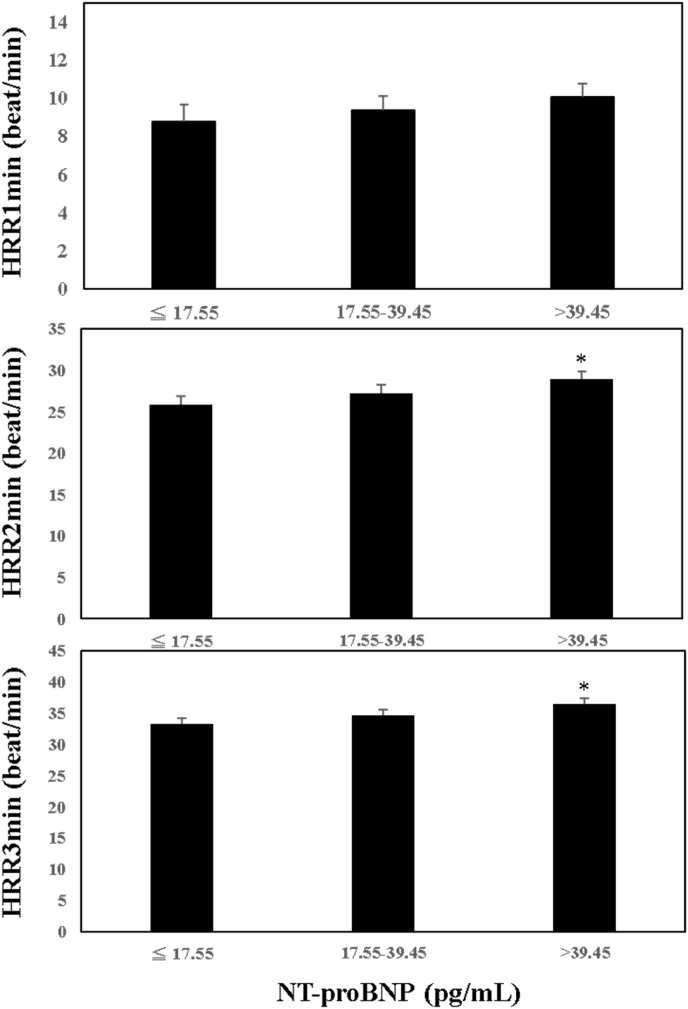
The adjusted values of HRR1 (1-min heart rate recovery), HRR2 and HRR3 among tertiles of N-terminal prohormone of brain natriuretic peptide (NT-proBNP). *, P < 0.05 as compared with the first tertile.

Fig. 2 demonstrates the adjusted values of different HRRs (fully adjusted) among tertiles of NT-proBNP. The HRR1 was not different among NT-proBNP tertiles (T1 to T3 = 8.76 ± 0.93, 9.35 ± 0.78, 10.06 ± 0.70 beat/min) while the HRR2 in the highest tertile was significantly higher than the HRR2 in the first tertile (26.69 ± 0.60 vs. 26.75 ± 0.51beat/min, P < 0.05). The HRR2 values between the first tertile and the second tertile and the HRR2 values between the second and the third tertile of the NT-proBNP were not significantly different. The result of HRR3 was similar to that of HRR2. The HRR3 in the highest tertile was significantly higher than the HRR3 in the first tertile (37.67 ± 0.76 vs. 34.70 ± 0.55 beat/min, P < 0.05). The HRR3 values between the first tertile and the second tertile and the HRR3 values between the second and the third tertile of the NT-proBNP were not significantly different.

## 4 Discussion

Studies have shown that the time course of HRR can be accurately modeled using a first-order exponential decay function [19,20]. This allows for the division of HRR into two distinct phases: the fast and slow. The fast phase corresponds to the initial minute of recovery (HRR1) and is characterized by a rapid and abrupt decrease in heart rate [21,22]. Conversely, the slow phase occurs after the fast phase, extending until the heart rate returns to its resting values [19,22,23]. Previous studies have established a correlation between HRR and autonomic activity, providing insight into the primary mechanisms driving HRR. Arai et al. observed a suppression of cardiac vagal modulation during exertion and its abrupt reactivation during the first minute of recovery [24]. In 1994, Imai et al. conducted a study using atropine and found a significant decrease in HRR within the first 30 s of recovery. Interestingly, blocking cardiac sympathetic activity did not impact HRR during this period. However, both vagal and sympathetic blockades did affect HRR at the 120-s after exercise [21]. In another study by Perini et al., they discovered a strong correlation between HRR and a decrease in plasma norepinephrine after the second minute of post-exercise, indicating that a decrease potentially influences the second phase of HRR in cardiac sympathetic activity [19]. In summary, these models suggest that the initial decrease in HRR is mainly due to vagal reactivation. However, after the first 2 min of recovery, HRR is influenced by both vagal reactivation and sympathetic withdrawal. Our study observed significant and positive correlations between NT-pro BNP and HRR2/HRR3, while the association between NT-proBNP and HRR1 was insignificant. These findings align with the autonomic mechanisms of heart rate regulation. BNP is known to have physiological effects, including inhibiting the SNS [6]. This provides a rationale for the observed relationship with HRR2 and HRR3, as these phases involve the reduction of HR through cardiac sympathetic withdrawal. However, there is also vagal modulation during this time. However, since BNP does not directly participate in vagal modulation, the statistical analysis did not reveal a significant association between NT-pro BNP and HRR1.

Our study also found significant correlations between NT-pro BNP and cardiovascular risk factors, including systolic and diastolic blood pressure and total cholesterol. Data from a Multi-Ethnic Study of Atherosclerosis showed that NT-proBNP within the lower (physiological) range was inversely associated with total cholesterol, triglyceride, and insulin resistance [25]. The same study also showed that these associations were blunted at higher (pathological) NT-proBNP levels [25]. The result reveals the potential relation between BNP and metabolic risks, which might partially explain the positive association between NT-proBNP and HRR. A study conducted in 2008 observed an inverse correlation between HRR and metabolic risks in healthy children and adolescents [26], which might contribute to the link between metabolic risks and autonomic nervous system functions [26,27]. In the same study, multiple linear regression analysis showed that the metabolic risks contribute more to the variation of the 2- or 3-min HRR compared with that of the 1-min HRR [26], with our study's results, suggesting a possible relation and coordinated interaction of metabolic risks and NT-proBNP, to HRR.

One major limitation of our study is that it is cross-sectional, making it impossible to determine causality between NT-proBNP, BNP, and HRR. Additionally, the submaximal treadmill test in NHANES may affect the HRR values.

In conclusion, we found that higher blood NT-proBNP level is associated with higher.

HRR2 and HRR3, indicating that BNP might have a direct beneficial effect in lowering sympathetic activity. Also, the BNP might indirectly exert its effect by improving metabolic risk factors.

## Credit author statement

Yi-Ting Lin: Writing – original draft, Conceptualization, Validation.Lian-Yu Lin: Formal analysis, Methodology.Kai-Jen Chuang: Writing- Reviewing and Editing.

## Declarations of interest

None.
